# PGM5 is a promising biomarker and may predict the prognosis of colorectal cancer patients

**DOI:** 10.1186/s12935-019-0967-y

**Published:** 2019-10-01

**Authors:** Yifan Sun, Haihua Long, Lin Sun, Xiujuan Sun, Liping Pang, Jianlin Chen, Qingqun Yi, Tianwei Liang, Yongqi Shen

**Affiliations:** 1 Department of Clinical Laboratory, Liuzhou Municipal Liutie Central Hospital, Liuzhou, Guangxi China; 2 Department of Endoscopy, Liuzhou Municipal Liutie Central Hospital, Liuzhou, Guangxi China; 3 Department of Pathology, Liuzhou Municipal Liutie Central Hospital, Liuzhou, Guangxi China; 4 Department of Gastroenterology, Liuzhou Municipal Liutie Central Hospital, Liuzhou, Guangxi China; 5 Department of Oncology, Liuzhou Municipal Liutie Central Hospital, No.14 Fei-e Road, Liuzhou, 545007 Guangxi China

**Keywords:** Phosphoglucomutase 5, Colorectal cancer, Overall survival

## Abstract

**Background:**

Phosphoglucomutase (PGM), a key enzyme in the metabolism of glucose-1-phosphate and glucose-6-phosphate, has been found to be associated with proliferation, invasion, and metastasis of cancer. However, the expression and function of PGM5 in colorectal cancer (CRC) remains unknown.

**Methods:**

We tested PGM5 mRNA and protein expression levels in 79 CRC tissue and their matched adjacent tissue samples by qRT-PCR and immunohistochemistry, respectively. Overall survival (OS) was estimated with the Kaplan–Meier method and compared between groups with the log-rank test. We performed multivariable Cox regression analyses to identify factors associated with CRC risk. The cell proliferation, migration and invasion abilities of CRC cells were detected by using CCK-8, Transwell migration and invasion assays, respectively.

**Results:**

The PGM5 protein levels expression in CRC tissues were significantly lower than those in the adjacent tissues (t = 5.035, *P *< 0.001), and Kaplan–Meier analysis indicated that low PGM5 expression were significantly associated with poor overall survival (*P *= 0.0069). Univariate and multivariate analyses demonstrated that PGM5 was an independent risk factor for overall survival (hazard ratio = 0.3951, *P *= 0.014). PGM5 overexpression significantly inhibited the proliferation, invasion and migration abilities of CRC cells. On the contrary, knockdown of PGM5 promotes the invasion and migration of CRC cells.

**Conclusions:**

PMG5 regulates proliferation, invasion, and migration in the CRC and decreased PGM5 is associated with poor prognosis. Therefore, PGM5 is a promising biomarker in CRC and decreased PGM5 may predict poor overall survival in patients with CRC.

## Background

Colorectal cancer (CRC) is the leading cause of death in the world and is a major public health problem with increasing incidence and mortality [1]. According to temporal trend analyses from 2000 to 2011, CRC remains a rapidly increasing cancer in China despite recent advances in its early detection and treatment [2].

Current primary treatment options depend on the stages of CRC. If patients are diagnosed at an early stage with small tumours, they have a better 5-year survival rate than those who are diagnosed at later stages, as most metastatic CRCs eventually cause death. However, many patients are already at an advanced stage at diagnosis in clinical practice. Therefore, reliable biomarkers are needed to help make clinical decisions throughout the patient’s disease trajectory to select the optimal oncological and surgical strategies.

Metastasis of cancer is associated with the tumour’s microenvironment. When energy metabolism remodelling occurs in the microenvironment, cell glycolysis activity is enhanced. Oxidative phosphorylation activity is then weakened, and the main energy source of the cell changes from oxidative phosphorylation to glycolysis, which is a new basic feature of tumour cells and tumours. Cell biosynthesis provides ‘raw materials’ and other functions, giving an evolutionary advantage to tumour cells [3]. Phosphoglucomutase (PGM) is a key enzyme in the metabolism of glucose-1-phosphate and glucose-6-phosphate [4], and PGM has been associated with the proliferation, invasion and metastasis of cancer [5, 6]. PGM5, also known as phosphoglycosidase-related protein (PGM-rp) or Aciculin, is localized on human chromosome 9 (9q21.11) and is represented by two closely related 60 kDa and 63 kDa subtypes [7]. PGM5 shows high expression in smooth muscle, skeletal muscle and heart muscle.

The Cancer Genome Atlas (TCGA) database suggested that PGM5 expression was significantly down-regulated in CRC. However, the expression and function of PGM5 in CRC remains unknown. Therefore, this study further confirmed the expression of PGM5 in CRC tissues and identified the function of PGM5 in CRC cell lines.

## Materials and methods

### The Cancer Genome Atlas data sets

TCGA-COAD and TCGA-READ using Ballgown cancerous and cancerous-adjacent datasets were compared to obtain the significant *P* value and the fold difference between the q-value and the gene fold change for differential gene screening. These values were obtained with the following conditions: a *P*-value less than 0.01, a q-value less than 0.05 and a fold change greater than 2. The results were normalized and log2-scaled using GEPIA’s online analysis tool (http://gepia.cancer-pku.cn/). The study was approved by the institutional review board of Liuzhou Municipal Liutie Central Hospital.

### Immunohistochemistry

The tissues’ PGM5 expression levels were evaluated by immunohistochemistry (IHC) using CRC samples (Superchip Biotech, Shanghai, China). Anti-Aciculin antibody (1:50; Santa Cruz) was applied as the first antibody, and the avidin–biotin–peroxidase method was used to visualize immunoreactivity. Haematoxylin was used as the counterstain.

The semi-quantitative scanning approach used to evaluate PGM5 expression was based on the scores of the positive cell percentage and positive cell staining intensity. The image analysis software Aperio Image scope version 11.1 (Aperio Technologies, Leicabiosystems; AT) was used to determine the immunostaining intensities. Additionally, integrated optical density (IOD) values were obtained for the tumour areas in each section, and these values were counted and measured using Image-Pro Plus v6.0 software (Media Cybernetics, Inc., Bethesda, MD, USA).

IHC assessment was conducted independently by two investigators, and immunoreactivity in the cytoplasm was quantified as described in a previous study [8]. The staining index was evaluated as follows: staining index = staining intensity (SI) × percentage of positive cells (PP). PP was scored as follows: 0 (˂ 5% positively stained cells), 1 (5 to 24% positively stained cells), 2 (25 to 49% positively stained cells), 3 (50 to 74% positively stained cells) and 4 (75 to 100% positively stained cells). SI was scored as follows: 0 (negative staining), 1 (weak staining), 2 (moderate staining) and 3 (strong staining). The PGM5 expression cut-off value was identified as the median IHC score.

### Cell lines and cell culture

The human CRC cell lines HT-29, DLD-1, HCT116, SW480, Caco-2 and LOVO, as well as the normal human colorectal cell FHC, were purchased from Hunan Fenghui Biotechnology Co., Ltd. (China). They were cultured in Dulbecco’s modified Eagle’s medium (DMEM; Gibco, USA) containing 10% foetal bovine serum (FBS; HyClone, USA) at 37 °C in a humidified chamber supplemented with 5% CO_2_.

### Real-time polymerase chain reaction test

The total RNA was extracted from the cell lines or tissues using Trizol reagent (Invitrogen, USA). Using a Reverse Transcription System Kit (Thermo #K1622, USA), cDNA was synthesized from the total RNA. The forward primers for PGM5 were as follows: 5′-TACAGCGTGGCGAAGACGGATAG-3′ and 5′-CTGCGTACAGTCTGAGGGTGGC-3′ for the reverse primers. PGM5 expression was analysed with the real-time polymerase chain reaction (RT-PCR) test using the SYBR Green PCR Kit (Thermo F-415XL, USA). β-Actin was used as a control, and the 2^−ΔΔCt^ method for the relative quantitation of gene expression was used to determine miRNA expression levels.

### Western blot analysis

Western blot analysis was performed for detection according to the standard protocol using peroxidase-conjugated secondary antibodies and the enhanced chemiluminescence system (Millipore, USA). The primary antibodies used were PGM5 (1:500; Novus, St. Louis, MO, USA) and beta-actin (1:2000; CST, Danvers, MA, USA, Catalogue #3700).

### Cell transfection

Lipofectamin2000 (Invitrogen) was used to transfect siRNA NC, siRNA-1 (TCAATGAAGGTCCCTGTAT), siRNA-2 (GGACTCAGGACGTTGCAAT) and siRNA-3 (GGTCTGGCTCTCCATTATT) into the high-expression PGM5 cell line according to the instructions. The pcDNA3.1-EGFP-PGM5 cloning plasmid was constructed and transfected into the low-expression PGM5 cell line. About 48 h after transfection, RT-PCR was used to detect whether the cell transfection was successful. The overexpression and knockdown efficiency were shown in Additional file 1: Figure S1.

### CCK-8 test

The cells were inoculated into a 96-well plate at 5 × 10^3^ cells for each well, and five parallel holes were set in each group. The cells were cultured for 12, 24, 36, 48, 72 and 96 h. CCK-8 was added to each well at 37 °C in a 5% CO_2_ incubator, and the cells were incubated in the dark for 1 to 4 h. A 450-nm wavelength microplate reader was used to measure the OD (Optical Density) value at the same time point, and the effect of cell growth was analysed using the measured OD value.

### Migration and invasion assay

In the migration assay, the control and transfected cells at 5 × 10^5^/well were seeded into the upper chambers containing DMEM with 1% FBS. About 600 µl of DMEM with 10% FBS was added to the lower chambers, and the cells were cultured at 37 °C in a CO_2_ incubator for 24 h. Afterwards, the upper chamber was removed, and the immigrated cells were carefully wiped from the membrane with a cotton swab. The cells were rinsed twice in a 37 °C pre-warmed PBS solution and were fixed with 4% paraformaldehyde for 30 min. Then, they were stained with haematoxylin for 5 min. Next, the cells were washed with distilled water and photographed under a microscope.

For the invasion assay, the upper chamber membranes were pre-coated with 50 µl of Matrigel (1.25 mg/ml). The control and transfected cells at 5 × 10^5^/well were seeded into the upper chambers containing DMEM with 1% FBS. The bottom chambers were filled with DMEM with 10% FBS containing chemokine and were cultured at 37 °C in a CO_2_ incubator for 24 h. Afterwards, the cells on the upper side of the upper-chamber membranes were removed. The invasion cells on the lower side of the membranes were fixed with 4% paraformaldehyde for 30 min and stained with haematoxylin for 5 min. The chambers were carefully removed from the upper chamber substrate; they were then mounted and counted under a microscope. A 200-fold light microscope was used to select and average five field counts randomly.

### Statistical analysis

All experiments were repeated three times independently, and the data were expressed as the mean ± SD. Statistical analysis was performed using SPSS 22.0 (SPSS Inc., Chicago, IL, USA) and GraphPad Prism 7 software. The relationship between PGM5 expression and clinicopathologic characteristics was measured using χ^2^ or Fisher’s exact tests. Overall survival (OS) was estimated using the Kaplan–Meier method. Univariate and multivariate survival analyses were performed using the Cox proportional hazard model with a forward stepwise procedure. A *P*-value less than 0.05 was considered statistically significant.

## Results

### Lower PGM5 expression in CRC

**Fig. 1:**
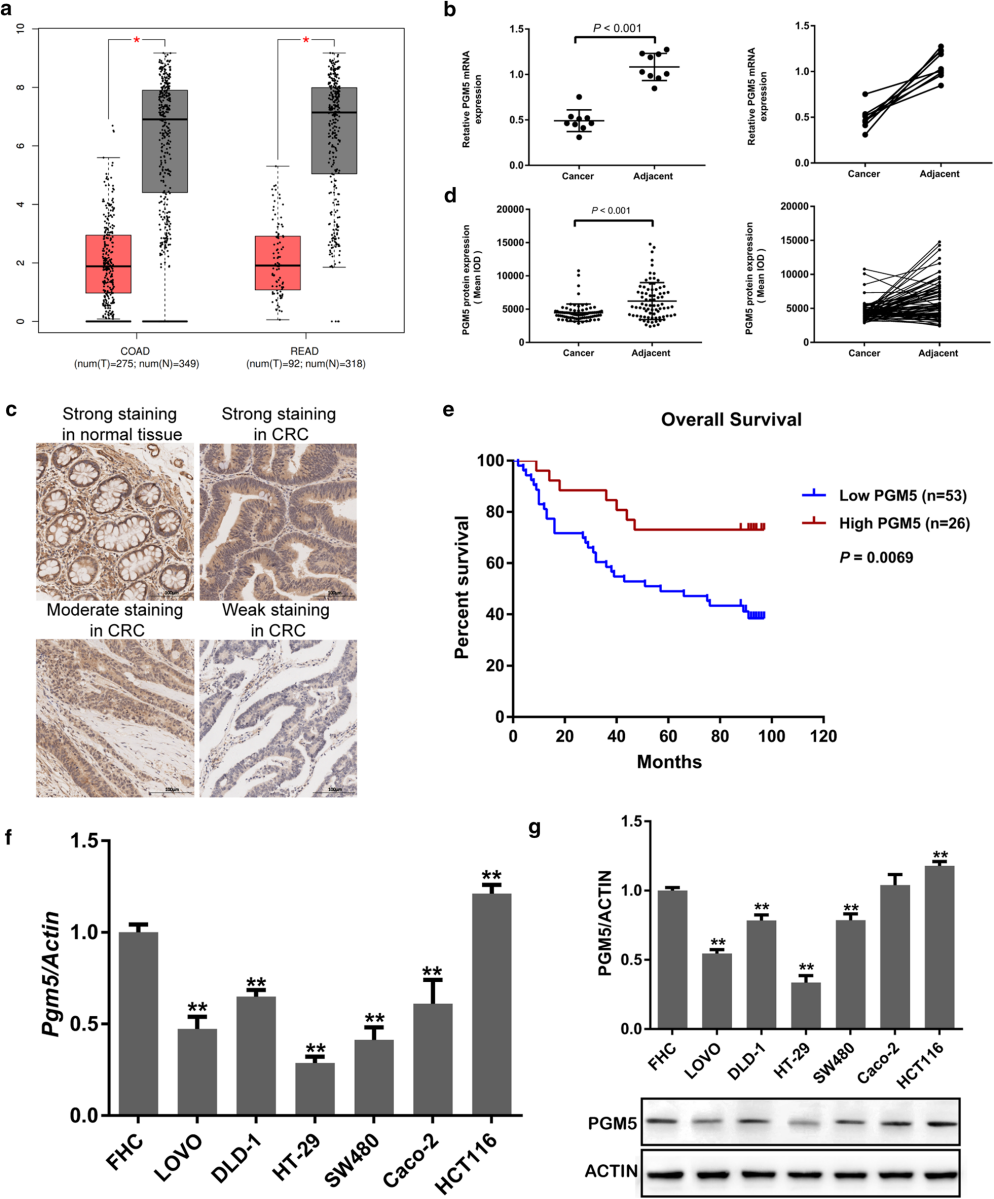
Downregulated PGM5 in CRC is associated with poor prognosis. **a** The PGM5 expression on Box Plotsacross all tumor samples and paired normal tissues in TCGA (Match TCGA normal and GTEx data, *P *< 0.001). Each dots represent expression of samples; **b** the expression level of PGM5 mRNA using RT-PCR; **c** representative images of PGM5 IHC staining in CRC and normal tissues (×200, scale bar 100 μm); **d** PGM5 protein expression in CRC tissues and adjacent tissues; **e** Kaplan–Meier analysis of the relationship between overall survival and PGM5 expression in patients with CRC; **f** The PGM5 expression in CRC cells. *PGM* phosphoglucomutase, *CRC* colorectal cancer, *TCGA* The Cancer Genome Atlas, *IOD* integrated optical density, *qRT-PCR* quantitative real-time PCR, *IHC* immunohistochemistry; **< 0.001

In TCGA database, PGM5 expression was found to be down-regulated significantly in gastrointestinal cancers, specifically COAD and READ (Fig. 1a). PGM5 expression in CRC was 0.17 times the expression in normal tissue (*P *< 0.0001). Nine pairs of clinical CRC tissues and matched adjacent tissues were selected to detect the expression levels of PGM5 miRNA and protein using RT-PCR. PGM5 miRNA expression in CRC tissues was significantly lower than in the adjacent tissues (t = 9.281, *P *< 0.001; Fig. 1b). To confirm these findings, PGM5 protein was examined using IHC in 79 CRC tissues (Fig. 1c), and IOD representing the expression level from 79 CRC tumours and the paired adjacent tissues was submitted to GraphPad Prism software. As shown in Fig. 1d, PGM5 expression was frequently lower in CRC tumour tissue than in the adjacent tissue (t = 5.035, *P *< 0.001). This suggested that the expression of PGM5 was down-regulated in CRC tissues.

### Decreased PGM5 in CRC associated with poor prognosis

To further evaluate PGM5 expression as a diagnostic marker for CRC, ROC curve analysis was performed, and the optimal cut-off value of PGM5 expression with the best discriminatory power was determined to be 6. Therefore, 79 immunostained CRC tissue samples were classified into two groups: high expression (index > 6, n = 26) and low expression (index ≤ 6, n = 53). As shown in Table 1, low PGM5 expression was significantly associated with lymph node metastasis (*P *= 0.041), clinical stage (*P *= 0.039) and poor survival (*P *= 0.005).

**Table 1:** Associations of PGM5 expression with clinicopathological factors in CRC patients

Variable	Number	PGM5 level		*P* value
		Low	High	
Age (years)				0.574
< 65	27	17 (21.5%)	10 (12.7%)	
≥ 65	52	36 (45.6%)	16 (20.3%)	
Gender				0.314
Male	39	25 (31.6%)	14 (17.7%)	
Female	40	28 (35.4%)	12 (15.2%)	
Tumor size (cm)				0.083
< 5	27	19 (24.1%)	8 (10.1%)	
≥ 5	52	34 (43.0%)	18 (22.8%)	
Lymph node metastasis				*0.041*
N0	48	28 (35.4%)	20 (25.3%)	
N1	22	16 (20.3%)	6 (7.6%)	
N2	9	9 (11.4%)	0 (24.1%)	
Clinical stage				*0.039*
I–II	48	20 (25.3%)	28 (35.4%)	
III–IV	31	6 (7.6%)	25 (31.6%)	
Survival				*0.005*
Live	40	21 (26.6%)	19 (24.1%)	
Dead	39	32 (40.5%)	7 (8.9%)	

Italic values indicate significance of *P* value (*P* < 0.05)

*PGM* phosphoglucomutase, *CRC* colorectal cancer

However, a statistically significant association between PGM5 expression and age, gender or tumour size was not found. Thus, the prognostic significance of PGM5 expression for CRC patients was further investigated. The Kaplan–Meier analysis using the most efficient cut-off value of PGM5 expression indicated that low PGM5 expression was significantly associated with poor overall survival compared with high PGM5 expression (*P *= 0.0069; Fig. 1e). Furthermore, univariate analysis showed that gender (*P *= 0.026), lymph node metastasis (*P *< 0.001), clinical stage (*P *= 0.001) and PGM5 expression (*P *= 0.001) were significant predictors of overall survival in CRC. Multivariate analysis indicated that only PGM5 expression (*P *= 0.015) was an independent predictive factor for poor outcomes in CRC patients (Table 2). These findings suggested that low PGM5 expression in CRC is correlated with poor prognosis of patients.

**Table 2:** Univariate and multivariate analysis

Clinical features	Univariate analysis		Multivariate analysis	
	HR (95% CI)	*P* value	HR (95% CI)	*P* value
Gender	1.749 (1.069–2.862)	*0.026*	1.535 (0.897–2.627)	0.118
Age	1.021 (0.996–1.047)	0.098		
Tumor size	1.416 (0.816–2.455)	0.216		
Lymph node metastasis	1.928 (1.419–2.621)	*< 0.001*	1.329 (0.704–2.507)	0.380
Clinical stage	2.393 (1.403–4.080)	*0.001*	1.387 (0.474–4.060)	0.551
PGM5 expression	0.302 (0.150–0.611)	*0.001*	0.395 (0.189–0.826)	*0.014*

Italic values indicate significance of *P* value (*P* < 0.05)

*PGM* phosphoglucomutase, *HR* hazard ratio, *CI* confidence interval

### PGM5 as a tumour suppressor in CRC

To evaluate the possible role of PGM5 in CRC and choose the CRC cell lines used for silencing or expressing PGM5, PGM5 expression was measured in six CRC cell lines as well as FHC cells. RT-PCR and Western blot analysis results indicated that PGM5 expression was highest in the HCT116 cell line (*P *= 0.005) and lowest in the HT-29 cell line (*P *< 0.001; Fig. 1f, g). The silencing effect of siRNA-3 was most significant among those of the control group and the negative control group (*P *= 0.001). Therefore, the HCT116 cell line was selected for silencing PGM5 using siRNA-3, and the HT-29 cell line was selected for overexpressing PGM5.

**Fig. 2:**
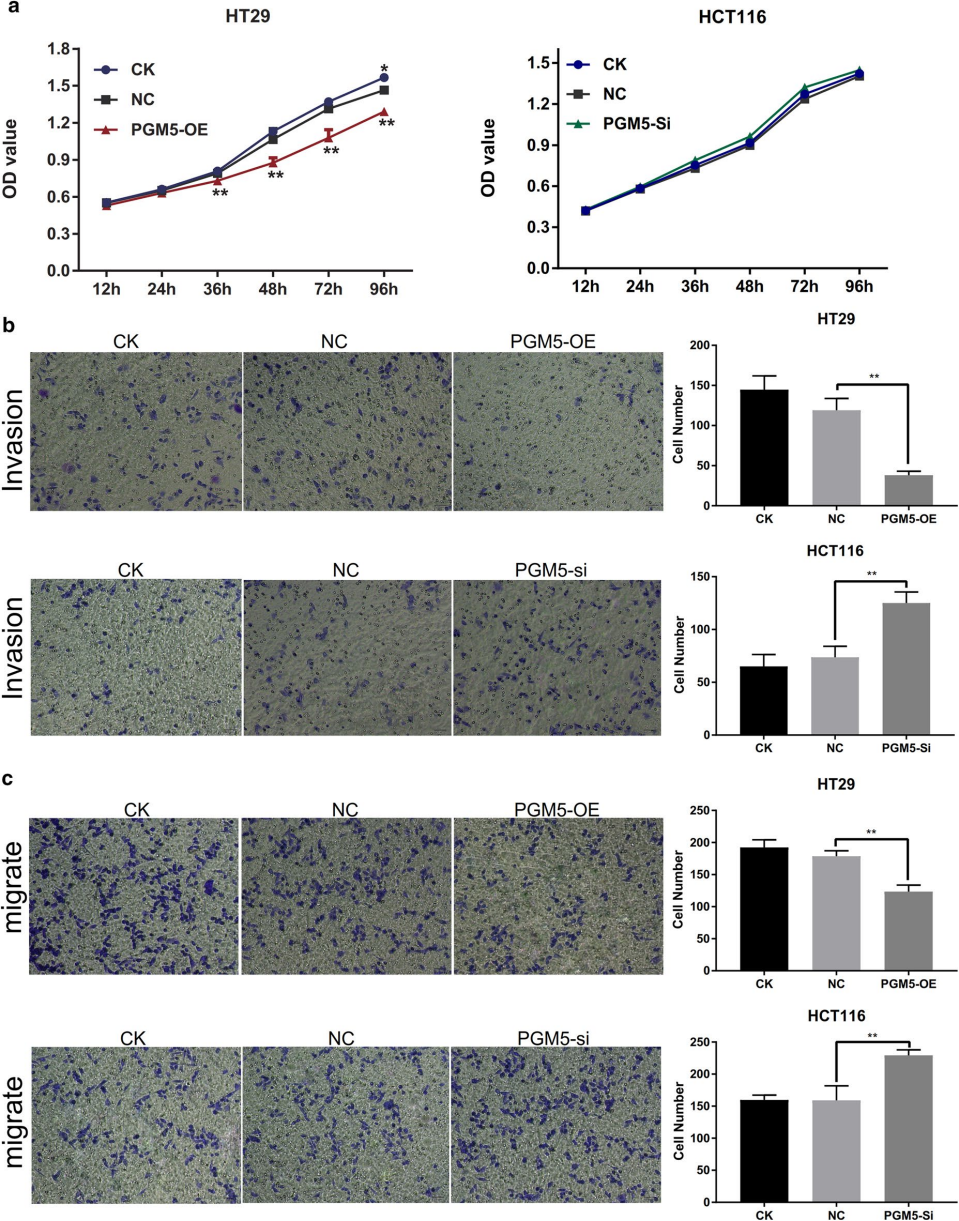
PGM5 is a tumor suppressor in CRC. **a** Upregulation of PGM5 remarkably inhibits the proliferation in HT29; **b** upregulation of PGM5 remarkably inhibits the invasion in HT29 and silence of PGM5 remarkably promotes the invasion in HCT116; **c** upregulation of PGM5 remarkably inhibits the migration in HT29 and silence of PGM5 remarkably promotes the migration in HCT116. *PGM* phosphoglucomutase, *CRC* colorectal cancer, *CK* normal cell group, *NC* negative control, *OE* over expression, *si* silence. **< 0.001

The up-regulation of PGM5 remarkably inhibited the cell’s proliferation (Fig. 2a), invasion (Fig. 2b) and migration abilities (Fig. 2c). PGM5 silencing did not significantly promote cell proliferation (Fig. 2a), but it remarkably promoted cell invasion and migration (Fig. 2b, c). These results suggested that PGM5 was a tumour suppressor in CRC.

## Discussion

The PGM superfamily contains five proteins: PGM1, PGM2, PGM2L1, PGM3 and PGM5. Although these five proteins share a homology in sequence coding, their substrates or functions are different and have different biological effects. For example, PGM1 gene defects damage hepatocyte homeostasis via the central metabolic pathway and then affect the growth of liver cancer cells [9]. PGM3, on the other hand, is an *N*-acetylglucosamine triphosphatase involved in the biosynthesis of the amino alanine, and it plays an anti-cancer role. For example, sulforaphane can reduce PGM3 expression in prostate cancer cells by inducing apoptosis [6]. PGM5 has a concentrated expression where muscles are connected [10], and it participates in myofibril formation, maintenance and transformation [11]. PGM5 is a binding partner for dystrophin; it binds to the N- and C-termini of dystrophin and reduces their expression [12]. In addition, PGM5 expression is associated with bipolar disorder [13] and heart disease [11]. However, the expression and function of PGM5 in CRC is still unknown.

Only a few studies have been conducted on the expression and role of PGM5 in cancer. In the gene chip of prostate cancer, the PGM5 antisense RNA1 (PGM5-AS1) expression was found to be down-regulated [14]. PGM5 expression was similarly down-regulated in bladder cancer tissues, but validation in 34 pairs of tissues showed that PGM5 expression in cancer and adjacent tissues was not significantly different (up-regulation in 15 cases and down-regulation in 19 cases) [15]. Therefore, PGM5 is not consistently expressed in cancer with different tissue types, and it may be related to the muscle content.

Recent studies have shown that PGM5 is down-regulated in colorectal adenomas or adenocarcinomas [16]. In this study, PGM5 was found to be decreased significantly in human CRC and was correlated with poor overall survival. Moreover, decreased PGM5 was an independent predictive factor for poor outcomes in CRC patients. Therefore, the data suggested that PGM5 could play an important role in the pathogenesis and development of CRC. Functionally, the up-regulation of PGM5 was found to inhibit the proliferation, migration and invasion of CRC cells, while the down-regulation of PGM5 exerted a contrary role. This strongly suggested that PGM5 was a novel tumour suppressor in CRC.

The tumour metastasis process is complex and involves many factors, including not only the changes in the tumour cells themselves but also the changes in the surrounding microenvironment. The tumour microenvironment, a key factor in tumour metastasis, is a dynamic network composed of tumour cells, the extracellular matrix and interstitial tissues [17, 18]. Rapid proliferation is one of the characteristics of tumour cells and requires a large amount of biological matter and energy; this proliferation changes the metabolic pattern and performs metabolic remodelling [19]. Therefore, reconstructing the energy metabolism system is also one of the characteristics of tumours [20].

PGM is a key enzyme in glucose metabolism, and PGM deficiency causes abnormal metabolism of glycogen, which leads to the emergence of various diseases [21]. Specifically, PGM5 encodes a glucose phosphate mutase, which plays a key role in carbohydrate metabolism [7]. Thus, this study hypothesized that a decrease in PGM5 could lead to an imbalance in energy metabolism, a metabolic remodelling of tumour cells and an abnormal increase in the aerobic glycolysis pathway in the tumour cells.

The glycolytic pathway not only provides a substrate for the biosynthesis of tumour cell proliferation but also produces a large amount of lactic acid. This lactic acid is released outside the cell, and this acidic environment promotes the cell’s proliferation, invasion, migration and so on [22, 23]. Due to the changes in tumour cells’ energy metabolism pathways, more tumour cells are needed to absorb enough glucose to meet their energy, biosynthesis and redox requirements. This, in turn, promotes tumorigenesis, tumour proliferation and invasion [3].

This study did face some limitations. These included the relatively small size of the study and relatively small statistical power. However, the results from TCGA database supported this study’s results.

## Conclusion

In conclusion, PGM5 was demonstrated to function as a tumour suppressor gene that is down-regulated in CRC. Moreover, lower expression of PGM5 was correlated with poorer prognosis in CRC patients. Functionally, the up-regulation of PGM5 was found to inhibit the proliferation, migration and invasion of CRC cells. Together, these findings suggested that PGM5 may serve as a potential novel predictive and therapeutic target for CRC.

## Supplementary information

**Additional file 1: Figure S1.** (A) The silence efficiency in HT29 cell line; (B) The over expression efficiency in HCT116 cell line. OE: over expression; si: silence **< 0.001.

## Data Availability

The datasets used in this study are available from the corresponding author upon reasonable request.

## Abbreviations

PGM: phosphoglucomutase
CRC: colorectal cancer
TCGA: The Cancer Genome Atlas
IOD: integrated optical density
qRT-PCR: quantitative real-time PCR
