# Supplementary figures and images for: PGM5 is a promising biomarker and may predict the prognosis of colorectal cancer patients
Source: Cancer Cell Int. 2019 Oct 1;19:253. doi: 10.1186/s12935-019-0967-y (PMC6771116; doi:10.1186/s12935-019-0967-y)

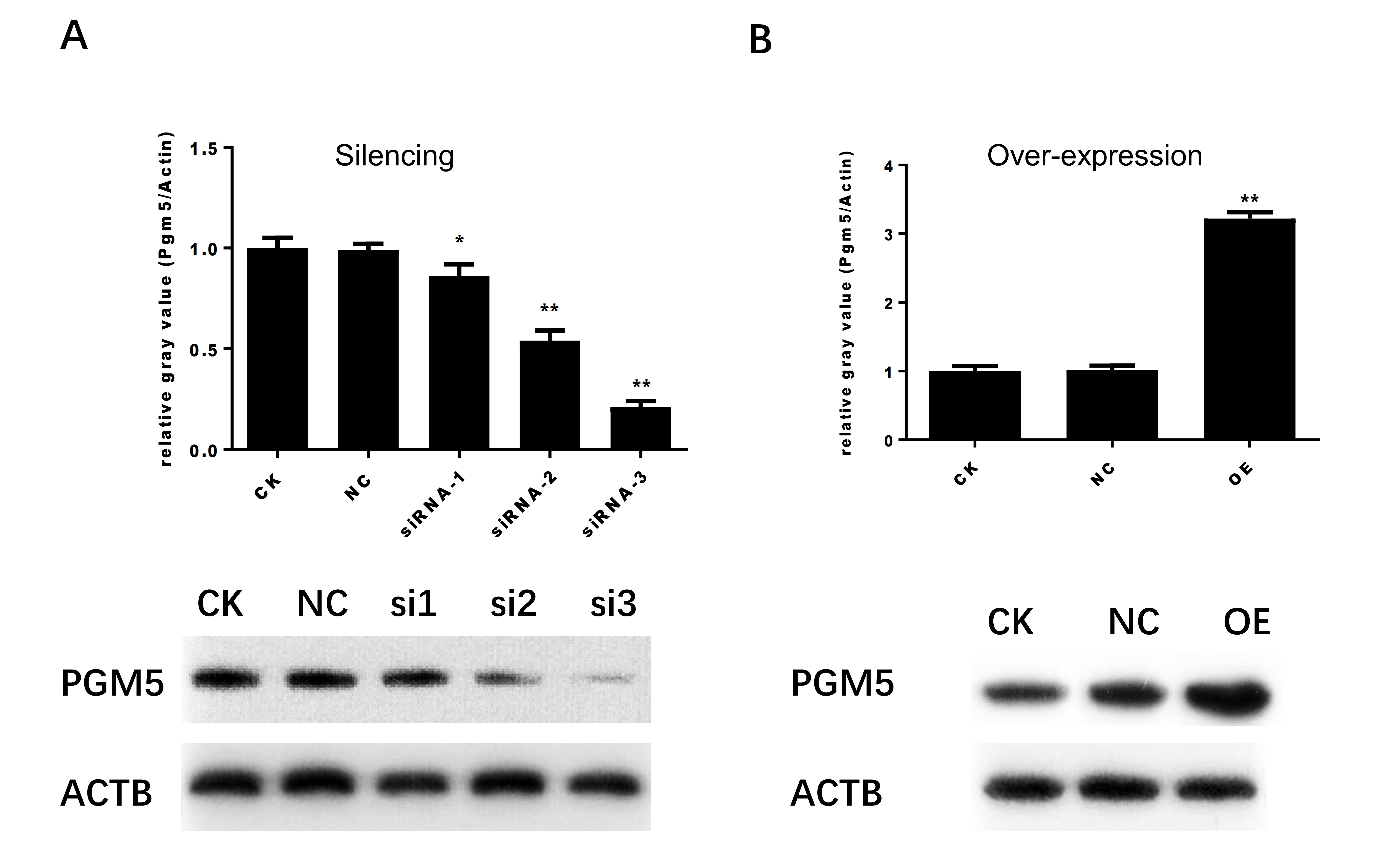

Supplement: Supplementary file 1 — Additional file 1: Figure S1. (A) The silence efficiency in HT29 cell line; (B) The over expression efficiency in HCT116 cell line. OE: over expression; si: silence **< 0.001. [file 12935_2019_967_MOESM1_ESM.tif]
